# Decreased Plasma Maresin 1 Concentration Is Associated with Diabetic Foot Ulcer

**DOI:** 10.1155/2020/4539035

**Published:** 2020-04-16

**Authors:** Tian Miao, Bangliang Huang, Niexia He, Lihua Sun, Guangsheng Du, Xiaoli Gong, Yong Xu, Yi Zheng, Hongting Zheng, Hua Qu

**Affiliations:** ^1^Department of Endocrinology, Translational Research of Diabetes Key Laboratory of Chongqing Education Commission of China, The Second Affiliated Hospital of Army Medical University, Chongqing, China; ^2^Department of Ultrasound, The Second Affiliated Hospital of Army Medical University, Chongqing, China; ^3^Department of General Surgery, The Second Affiliated Hospital of Army Medical University, Chongqing, China; ^4^Department of Diabetes and Endocrinology, Affiliated Hospital of Southwest Medical University, Luzhou, Sichuan, China

## Abstract

**Aims:**

To assess the maresin 1 (MaR1) contents in type 2 diabetic patients with or without diabetic foot ulcer and to analyze the association of MaR1 concentrations with several metabolism-related parameters.

**Methods:**

Plasma MaR1 concentrations were analyzed in 96 subjects with normal glucose tolerant (NC, *n* = 43), type 2 diabetes (T2DM, *n* = 40), or diabetic foot ulcer (DFU, *n* = 13). The intravenous glucose tolerance test (IVGTT) and biochemical parameters were measured in all participants.

**Results:**

Plasma MaR1 concentrations were significant decreased in type 2 diabetes patient with or without DFU compared with NC (both *P* < 0.001) and were lowest in DFU patients among these 3 groups. (DFU vs. T2DM, *P* < 0.05). Plasma MaR1 concentrations were negatively correlated with BMI, waist circumference (Wc), waist hip ratio (WHR), systolic blood pressure (SBP), diastolic blood pressure (DBP), LDL-c, FPG, 2hPG, HbA1c, and homeostasis model assessment for insulin resistance (HOMA-IR) (all *P* < 0.05) and were positively correlated with HDL-c, acute insulin response (AIR), area under the curve of the first-phase (0-10 min) insulin secretion (AUC), and homeostasis model assessment for beta-cell function (HOMA-*β*) (all *P* < 0.05). After adjusting for age and sex, Wc, WHR, TG, FPG, 2hPG, HbA1c, HOMA-IR, AIR, AUC, and HOMA-*β* remain statistically significant (all *P* < 0.05).

**Conclusions:**

Plasma MaR1 concentration were decreased in T2DM with or without DFUs and were the lowest in DFU patients. The decreased plasma MaR1 strongly associated with obesity, impaired glucose and lipid metabolism, reduced first-phase of glucose-stimulated insulin secretion, and enhanced insulin resistance.

## 1. Introduction

Diabetic foot ulcer (DFU), exhibited as delayed healing of wounds in feet and lower limbs, is one of the main causes of disability and amputation of diabetic patients and is also a major public health problem that causes global burden on society and economy. It is estimated that DFU affect between 9.1 and 26.1 million people worldwide, and there is one case of diabetic amputation every 30 seconds in the world [1, 2]. The 5-year mortality rate is about 44% and may be as high as 70% when patients have a related amputation [3, 4]. Multiple factors have been identified as contributors to the pathophysiology of DFU. Among these factors, excessive inflammatory cytokine accumulation lead to sustained inflammatory responses was considered as one of the most important reason [5, 6]. Although diabetes is deeply linked to the chronic inflammatory response, the mechanism by which inflammatory cytokine accumulation resulting in impaired wound healing remains unclear.

Maresin (MaR) family is a newly described macrophage-derived mediator of inflammation resolution [7]. MaR1 is the first reported member of this family and shows broad anti-inflammatory actions under physiologic conditions in endothelial cells and vascular smooth muscle cells [8], human periodontal ligament cells [9], and has been reported to have benefits on multiple diseases, such as acute pancreatitis [10], neurocognitive disorders [11], and inflammatory arthritis [12]. More important, recent studies also revealed that MaR1 play roles in obesity [13] and nonalcoholic steatohepatitis [14], indicating a strong association between MaR1 and metabolic diseases. Based on its inflammation and metabolism regulation role, we therefore speculate that MaR1 might also have an effect in diabetic wound healing.

In this report, we recruited diabetic patients with or without foot ulcer (DFU) to analyze the concentration of plasma MaR1 and other metabolic parameters and to explore the potential relationship of them.

## 2. Methods

### 2.1. Subjects

A total of 115 subject participant in our study, and after screening, 40 patients with newly diagnosed T2DM (without foot ulcer), 13 DFU patients (SINBAD score ranging from 2 to 4), and 43 healthy subjects (NC) were recruited in this study. The T2DM were diagnosed by oral glucose tolerance tests (OGTT) according to the American Diabetes Association diagnostic criteria (2017) [15]. DFU diagnosed according to the International Working Group on Diabetic Foot (IWGDF) definition [16]. The exclusion criteria are as follows: (1) smoking and drinking history, (2) acute complications of diabetes, (3) hepatic or renal disease and systemic corticosteroid treatment, and (4) women who were currently pregnant and breastfeeding. All experimental protocols were approved by the Ethics Committee of Xinqiao Hospital, Third Military Medical University and registered online (Clinical trial register no. ChiCTR-ROC-17010719). This study was conducted in accordance with the Helsinki Declaration.

### 2.2. Clinical Evaluation of Subjects

Clinical parameters such as height, body weight, waist circumferences (Wc), hip circumferences (Hc), and systolic and diastolic blood pressure (SBP and DBP) were measured according to our previous protocols [17, 18]. Body weight was measured to an accuracy of +0.2 kg. Height, waist, and hip circumferences were measured to minimum recorded unit 0.1 cm. Systolic and diastolic blood pressure was measured twice by a standard mercury manometer with the subjects seated. Body mass index (BMI) and waist to hip ratio (WHR) were calculated according to a standardized protocol.

Overnight fasting blood samples were collected and analyzed as described by our previous studies [17, 18]. All blood samples were separated within 1 hour and then frozen at -80°C until use in this study, all within 3 months. Hemoglobin A1c (HbA1c) was measured by isoelectric focusing. Triglyceride (TG), total cholesterol (TC), high-density lipoprotein cholesterol (HDL-c), low-density lipoprotein cholesterol (LDL-c), and high-sensitive C-reactive protein (hsCRP) were detected by biochemical autoanalyzer (Beckman CX-7 Biochemical Autoanalyser, Brea, CA, USA).

### 2.3. Oral Glucose Tolerance Test (OGTT)

After an 8- to 10-h overnight fasting, OGTT was performed in all subjects. A glucose solution (contain 75 g glucose) was ingested within 5 min by each subject, and blood samples were obtained before (0 min) and at 120 min for glucose and insulin assessments.

### 2.4. Intravenous Glucose Tolerance Test (IVGTT)

All subjects suggested to a diet containing at least 150 g of carbohydrate/day for 3 days before the test. After an 8- to 10-h fasting, IVGTT was performed in all subjects. A 50% glucose solution was infused as a square wave bolus over 3 min with a dose of 300 mg/kg body weight glucose (maximum dose 35 g) for each subject, and blood samples were obtained before (0 min) and at 3, 5, 8, and 10 min after glucose infusion for glucose and insulin determinations.

### 2.5. Assessment of Plasma MaR1 Levels

Overnight fasting blood samples were collected according to the requirement of the specimens, EDTA were chosen as anticoagulant, mixed 10-20 minutes, centrifuged 20 minutes (2000-3000 revolutions per minute), and carefully collected the supernatant [19]. The plasma MaR1 concentrations were determined by commercial ELISA kits according to the manufacturers' instructions (Human ELISA kit, Senbeijia, NanJing, China). The absorbance (OD value) was measured by microplate at the wavelength of 450 nm, and the content of MaR1 in the sample was calculated by standard curve [20]. All samples were run in duplicate and repeated if there was a >15% difference between duplicates. Seven samples required rerun, and the redetected results were used for further analyses. The detection range is 125 to 8,000 pg/mL, and intra-assay coefficient of variance (CV) was 10% and interassay CV of 12%. No significant cross-reactivity or interference was observed.

### 2.6. Related Calculation Formula

Body mass index (BMI) formula is weight in kilograms divided by height in meters squared. The area under curve (AUC) of the first phase insulin secretion, i.e., 0 to 10 min of glucose-stimulated insulin secretion, was calculated by the irregular trapezoid formula. Acute insulin response (AIR) was calculated as the mean plasma insulin concentration of 3 and 5 min after glucose infusion. Homeostasis model assessment of insulin resistance (HOMA-IR) was calculated by fasting insulin (*μ*U/mL) X fasting plasma glucose (mmol/L)/22.5, and homeostasis model assessment of *β*-cell function was calculated as 20 X fasting insulin (*μ*U/mL)/[fasting plasma glucose (mmol/L) - 3.5].

### 2.7. Statistical Analyses

All statistical analyses were conducted by the SPSS software (IBM, Armonk, NY, version 19.0). The normal distribution of the data was detected using Kolmogorox-Smirnov test. Several variables were showed skewed distribution and were logarithmically transformed into normal distribution before statistical analysis. One-way ANOVA with Tukey's post hoc test was performed for multiple comparisons. Interrelationships between variables were estimated using Pearson's correlation coefficient with or without adjusting for age and sex. Multivariate logistic regression analyses were used to analyze the association between plasma MaR1 concentrations and T2DM without or with DFU. *P* *values* < 0.05 were regarded as statistically significant.

## 3. Results

### 3.1. Clinical and Laboratory Characteristics of Study Participants

As shown in Table 1, the average age of our study was 54.2 + 11.2 years, and 34 (35.4%) males and 62 (64.6%) females were included. BMI were significantly higher in T2DM and DFU patients compared with normal control (NC) subjects (*P* < 0.05 and *P* < 0.01), but there was no significant difference between T2DM and DFU groups (*P* > 0.05). Moreover, waist circumferences and WHR were significantly higher in DFU patients compared with T2DM (*P* < 0.01) and NC subjects (*P* < 0.01), while no significant difference was found between T2DM and NC (*P* > 0.05) groups. SBP has no significant differences among these 3 groups, while DBP were the highest in DFU group compared with both T2DM and NC group (both *P* < 0.01).

As for glucose-metabolic parameters, the fasting plasma glucose (FPG), 2 h postchallenge plasma glucose (2hPG), HbA1c, and HOMA-IR were significant higher in T2DM and DFU patients compared with NC subjects and were the highest in DFU (*P* < 0.01 or *P* < 0.05, Table 1) group. Moreover, fasting serum insulin (FINS) was significantly increased in DFU compared with NC subjects (*P* < 0.05). Meanwhile, a *β*-cell function indicator-HOMA-*β* was found to significantly reduced in DFU and T2DM patients compared to NC subjects (*P* < 0.01). Consistent with this, our IVGTT test also found the levels of AUC and AIR were significantly reduced in DFU and T2DM patients compared to NC subjects (*P* < 0.01 or *P* < 0.05).

Previous preclinical studies have reported that mice treated with MaR1 showed altered serum LDL/VLDL levels, and it also exerts potent anti-inflammatory and proresolution activities under both physiologic and disease conditions [9, 21, 22]. Thus, we assessed lipid-metabolic parameters and inflammatory factors in our subjects. As shown in Table 1, HDL-c was significantly decreased in DFU patients compared with NC subjects (*P* < 0.05). TG was significantly higher in T2DM than NC subjects (*P* < 0.01), but there was no significant difference between DFU and NC (*P* > 0.05). While TC, LDL-c, and hs-CRP levels have no significant differences among these 3 groups (*P* > 0.05).

### 3.2. Circulating MaR1 Levels in Different Groups

**Figure 1:**
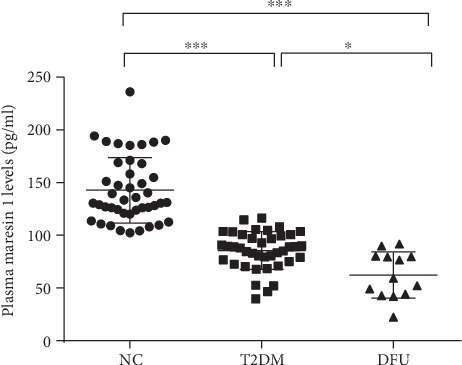
Scatter plot of plasma maresin 1 concentrations in subjects with different glucose tolerances. Each data point represents a plasma sample, the horizontal middle line in each data set represents the mean, and the limits of the vertical lines represent the SD. One-way ANOVA with Tukey's post hoc test was performed for multiple comparisons. ∗∗∗*P* < 0.001 compared with NC; ∗*P* < 0.05 compared with T2DM.

Plasma MaR1 concentrations were significant decreased in T2DM patients with or without DFU compared with NC subjects (both *P* < 0.001, Figure 1) and were least in DFU patients (compared with T2DM group, *P* < 0.05, Figure 1). These were no significant differences of MaR1 concentrations between men and women (105.64 + 45.31 vs. 109.34 + 38.46 *pg*/*mL*, *P* = 0.673).

### 3.3. Association of Plasma MaR1 Levels with Metabolic Related Parameters

Next, we investigate the association of circulating MaR1 concentrations and metabolic-related parameters. Plasma MaR1 concentrations were negatively correlated with BMI, Wc, WHR, SBP, DBP, LDL-c, FPG, 2hPG, HbA1c, and HOMA-IR (all *P* < 0.05, Table 2) and were positively correlated with HDL-c, AIR, AUC, and HOMA-*β* (all *P* < 0.05, Table 2). After adjusting for age and sex, MaR1 remains statistically negatively correlated with Wc, WHR, TG, FPG, 2hPG, HbA1c, and HOMA-IR and positively associated with AIR, AUC, and HOMA-*β* (all *P* < 0.05, Table 2). These finding indicated a strong correlation between MaR1 and obesity, lipids and glucose metabolism, and insulin resistance and secretion.

Further, multivariate logistic regression analysis revealed that decreased plasma MaR1 concentrations were significantly associated with T2DM without or with DFU after controlling for age, sex, BMI, WHR, blood pressure, and lipid profiles (odds ratio, 0.042 and 0.011, 95% confidence interval 0.006-0.29 and 0.007-0.15, both *P* < 0.001).

## 4. Discussion

MaR1, a potent proresolving lipid mediator, has been demonstrated to correlate with inflammation resolution, tissue homeostasis, and tissue regeneration [7]; these physiologic functions were deeply associated with wound healing process. Thus, we speculated that MaR1 might be associated with impaired wound healing process in diabetic patients, which has not been explored before. In our study, we found plasma MaR1 concentrations were significantly decreased in T2DM subjects with or without DFU compared with those in the NC group. Notably, these DFU patients showed the lowest MaR1 level in these 3 groups. Additionally, correlation analysis demonstrated that plasma MaR1 levels were significantly correlated with parameters regarding obesity, glucose metabolism, lipid profiles, insulin secretion, and insulin resistance.

MaRs are newly discovered macrophage inflammatory mediators, and these new mediators are biosynthesized in macrophages by 14 lipid oxidation of docosahexaenoic acid, producing 14 hydroperoxydocosac-4*Z*, 7*Z*, 10*Z*, 12*E*, 16*Z*, 19*Z* hexanoic acid (14 HPDHA). The latter is further transformed by 13 (14)-epoxidation, which is an important process of biosynthesis of 7*R*, 14 dihydroxydocosa-4*Z*, 8*E*, 10*E*, 12*Z*, 16*Z*, 19*Z* hexanoic acid, which are named as MaR1 [23, 24]. Previous in vitro and in vivo evidences have appeared indicating that MaR1 exerts potent anti-inflammatory and proresolution activities under both physiologic and disease conditions. It is reported that MaR1 can exert protective actions in murine models of colitis [22], regulates autophagy and inflammation in human periodontal ligament cells [9], attenuates inflammation in vascular smooth muscle and endothelial cells, and mitigates LPS-induced acute lung injury [25]. Inflammation and its resolution are also critical to the processes of wound healing. Cutaneous wound healing is a complex process that has been divided into three phases, including inflammation, tissue formation, and tissue remodeling [26]. After injury, inflammation begins immediately marked by infiltration of neutrophils and macrophages [26, 27]. The appropriate response of inflammation promotes debridement of devitalized tissues and to combat invading microbes by releasing antimicrobial peptides, eicosanoids, proteases, and neutrophil extracellular traps [27, 28]. However, excessive inflammatory responses delayed healing process by leading to tissue destruction, which is exactly happened in chronic nonhealing or slow-healing wounds, including DFUs [5, 29]. Our study found a significant negative correlation of MaR1 and diabetes with or without DFUs, and more importantly, MaR1 was lower in DFUs compared with these diabetic patients without DFUs. Consistently, lipid factors have been reported to influence wound healing in diabetic patients by others. Zubair et al. [30] conducted a meta-analysis reported that adiponectin which is the most abundant adipocytokine, associated with foot ulcers pathogenesis by inflammatory and microvascular mechanisms, and Wang et al. [31] also reported that ganglioside GM3, a sialylated membrane-based glycosphingolipid, can affect wound healing in diabetic mice by activating IGF-1 and insulin receptors.

Recently, Luiza et al. found the plasma MaR1 levels were significantly negatively correlated to BMI in preeclampsia women [20]. Our results also showed a significant negative relation between BMI, WHR, and MaR1 levels in all our participants, while after controlling for both BMI and WHR, MaR1 remains statistically associated with both diabetes with or without DFUs, suggested that obesity might not be a mediator of this association.

Previous study [21] reported that MaR1 could affect metabolic pathway in CLP-induced septic mice. They found that the levels of LDL/VLDL, glucose, isoleucine, and acetone levels were reduced in serum of CLP-induced septic mice. Of note, MaR1 treatment markedly increased these metabolites in serum, suggested that MaR1 could regulate lipid metabolism. Our results also showed a significant negative relationship between TG and MaR1 levels, supported that MaR1 affect lipid metabolism.

Previous animal study showed that MaR1 treatment could improve the insulin tolerance test of ob/ob mice and increased Akt and AMPK phosphorylation in white adipose tissue [13]. Thus, the author suggested that the treatment with MaR1 might be a useful therapy to improve insulin sensitivity in murine models of obesity [13]. Consistent with this, our population study showed a significant negative relation between MaR1 and insulin resistance in diabetic patients. Notably, after IVGTT test, we also found a positive correlation of MaR1 and glucose-stimulated insulin secretion, indicating a potential role of MaR1 in *β*-cell function and suggesting further exploration are needed in future studies.

The current study has some limitations needed to be addressed. First, the causality between plasma MaR1 levels and DFU or T2DM cannot be established in our study due to the cross-sectional design. Previous animal studies found that MaR1 can attenuate inflammatory signaling pathways in vascular smooth muscle and endothelial cells after vascular injury. Thus, we speculated that the decreased MaR1 in T2DM and DFU patients might be a cause of lack of resolution of inflammation. However, we also cannot rule out the possibility that the decreased MaR1 is a result of DFU; further studies are needed to clarify this. Second, the sample size of DFU groups were relatively small; thus, a selection bias might exist in current design. Third, medicine uses which might have influence on the plasma MaR1 levels are not analyzed in this study. Finally, the plasma concentration of MaR1 may also be influenced by its catabolism, which was not assessed in our study.

In conclusion, our study demonstrated that the plasma MaR1 levels were significantly decreased in diabetic patients with or without DFUs compared with healthy subjects and were the lowest in DFU patients among these 3 groups. The concentrations of plasma MaR1 correlated closely with multiple clinical parameters involving obesity, glucose and lipid metabolism disorders, and insulin secretion and resistance. Thus, the circulating MaR1 may be associated with diabetes with or without foot ulcers.

## Figures and Tables

**Table 1:** Clinical and laboratory characteristics of the study participants.

	NC	T2DM	DFU
Sex (M/F)	43 (12/31)	40 (14/26)	13 (8/5)
Age (year)	52.98 + 11.79	54.58 + 9.73	56.62 + 10.11
BMI (kg/m^2^)	21.85 + 3.41	23.80 + 2.95^a^	25.56 + 3.50^b^
Wc (cm)	79.81 + 9.40	84.01 + 7.25	94.27 + 8.68^bd^
WHR	0.85 + 0.06	0.87 + 0.07	0.94 + 0.05^bd^
SBP (mm hg)	121.63 + 13.72	125.83 + 13.93	130.08 + 15.06
DBP (mm hg)	76.17 + 9.20	76.30 + 9.43	85.38 + 9.35^bd^
TC (mmol/L)	4.47 + 0.98	4.68 + 1.03	4.34 + 0.69
TG (mmol/L)	1.23 + 0.52	1.95 + 1.41^b^	1.45 + 0.52
HDL-c (mmol/L)	1.54 + 0.56	1.32 + 0.31	1.16 + 0.17^a^
LDL-c (mmol/L)	2.37 + 0.99	2.82 + 0.76	2.61 + 0.72
hsCRP (mg/L)	1.55 + 3.68	0.94 + 0.79	1.53 + 0.79
FPG (mmol/L)	5.16 + 0.35	7.67 + 0.26^b^	9.17 + 2.38^bd^
2hPG (mmol/L)	4.70 + 0.60	7.92 + 1.15^b^	10.43 + 3.03^bd^
HbA1C (%/mmol/mol)	5.52 + 0.36/36	6.01 + 0.42/42^a^	8.82 + 2.29/73^bd^
FINS (mU/L)	5.75 + 3.89	6.97 + 3.77	8.27 + 5.49^a^
AUC	498.95 + 481.04	164.30 + 99.48^a^	100.82+151.02^b^
AIR	62.89 + 61.81	17.54 + 10.63^a^	10.89 + 17.45^b^
HOMA-IR	1.33 + 0.94	2.08 + 1.15^a^	3.25 + 2.01^bd^
HOMA-*β*	70.92 + 45.79	43.76 + 22.63^b^	35.16 + 31.84^b^
Insulin treatment (/%)	0	0	8/61.54
OAD treatment (/%)	0	0	5/38.46

Data are presented as *means* + *SD*. NC: normal control; T2DM: newly diagnosed type 2 diabetes; DFU: diabetic foot ulcer; BMI: body mass index; Wc: waist circumference; WHR: waist hip ratio; SBP: systolic blood pressure; DBP: diastolic blood pressure; TC: total cholesterol; TG: triglyceride; HDL-c: high-density lipoprotein-cholesterol; LDL-c: low-density lipoprotein-cholesterol; hsCRP: hypersensitive C reactive protein; FPG: fasting plasma glucose; 2hPG: 2 h postchallenge plasma glucose; FINS: fasting serum insulin; AUC: area under the curve of the first-phase (0-10 min) insulin secretion; AIR: acute insulin response; HOMA-IR: homeostasis model assessment for insulin resistance; HOMA-*β*: homeostasis model assessment for beta-cell function; OAD: oral antidiabetic drug. ^a^*P* < 0.05 compared with NC; ^b^*P* < 0.01 compared with NC; ^d^*P* < 0.01 compared with T2DM.

**Table 2:** Pearson correlation coefficient of variables associated with circulating concentration in study population.

	Plasma maresin 1		Plasma maresin 1 (age- and sex-adjusted)	
	*r*	*P-*value	*r*	*P-*value
Age (year)	0.066	0.524	—	—
Sex (M/F)	0.044	0.673	—	—
BMI (kg/m^2^)	-0.432	**<0.001**	-0.303	0.061
Wc (cm)	-0.396	**<0.001**	-0.378	**0.018**
WHR	-0.346	**<0.001**	-0.471	**0.002**
SBP (mm hg)	-0.212	**0.040**	-0.255	0.117
DBP (mm hg)	-0.238	**0.021**	-0.258	0.113
TC (mmol/L)	-0.107	0.299	0.191	0.244
TG (mmol/L)	-0.185	0.072	-0.331	**0.039**
HDL-c (mmol/L)	0.223	**0.029**	0.201	0.219
LDL-c (mmol/L)	-0.230	**0.024**	0.127	0.442
hsCRP (mg/L)	0.056	0.598	0.224	0.170
FPG (mmol/L)	-0.575	**<0.001**	-0.610	**<0.001**
2hPG (mmol/L)	-0.566	**<0.001**	-0.652	**<0.001**
HbA1_C_ (%)	-0.487	**<0.001**	-0.518	**<0.001**
FINS (mU/L)	-0.173	0.092	-0.144	0.382
AUC	0.463	**0.001**	0.461	**0.003**
AIR	0.481	**<0.001**	0.484	**0.002**
HOMA-IR	-0.332	**<0.001**	-0.317	**0.050**
HOMA-*β*	0.291	**0.004**	0.365	**0.022**

BMI: body mass index; Wc: waist circumference; WHR: waist hip ratio; SBP: systolic blood pressure; DBP: diastolic blood pressure; TC: total cholesterol; TG: triglyceride; HDL-c: high-density lipoprotein-cholesterol; LDL-c: low-density lipoprotein-cholesterol; hsCRP: hypersensitive C-reactive protein; FPG: fasting plasma glucose; 2hPG: 2 h postchallenge plasma glucose; FINS: fasting serum insulin; AUC: area under the curve of the first-phase (0-10 min) insulin secretion; AIR: acute insulin response; HOMA-IR: homeostasis model assessment for insulin resistance; HOMA-*β*: homeostasis model assessment for beta-cell function.

## Data Availability

The clinical characteristic data of all human subjects used to support the findings of this study are included within the article.
